# microPIR: An Integrated Database of MicroRNA Target Sites within Human Promoter Sequences

**DOI:** 10.1371/journal.pone.0033888

**Published:** 2012-03-16

**Authors:** Jittima Piriyapongsa, Chaiwat Bootchai, Chumpol Ngamphiw, Sissades Tongsima

**Affiliations:** Genome Institute, National Center for Genetic Engineering and Biotechnology, Pathumthani, Thailand; Georgia Institute of Technology, United States of America

## Abstract

**Background:**

microRNAs are generally understood to regulate gene expression through binding to target sequences within 3′-UTRs of mRNAs. Therefore, computational prediction of target sites is usually restricted to these gene regions. Recent experimental studies though have suggested that microRNAs may alternatively modulate gene expression by interacting with promoters. A database of potential microRNA target sites in promoters would stimulate research in this field leading to more understanding of complex microRNA regulatory mechanism.

**Methodology:**

We developed a database hosting predicted microRNA target sites located within human promoter sequences and their associated genomic features, called microPIR (microRNA-Promoter Interaction Resource). microRNA seed sequences were used to identify perfect complementary matching sequences in the human promoters and the potential target sites were predicted using the RNAhybrid program. >15 million target sites were identified which are located within 5000 bp upstream of all human genes, on both sense and antisense strands. The experimentally confirmed argonaute (AGO) binding sites and EST expression data including the sequence conservation across vertebrate species of each predicted target are presented for researchers to appraise the quality of predicted target sites. The microPIR database integrates various annotated genomic sequence databases, e.g. repetitive elements, transcription factor binding sites, CpG islands, and SNPs, offering users the facility to extensively explore relationships among target sites and other genomic features. Furthermore, functional information of target genes including gene ontologies, KEGG pathways, and OMIM associations are provided. The built-in genome browser of microPIR provides a comprehensive view of multidimensional genomic data. Finally, microPIR incorporates a PCR primer design module to facilitate experimental validation.

**Conclusions:**

The proposed microPIR database is a useful integrated resource of microRNA-promoter target interactions for experimental microRNA researchers and computational biologists to study the microRNA regulation through gene promoter. The database can be freely accessed from: http://www4a.biotec.or.th/micropir.

## Introduction

MicroRNAs (miRNAs) are small ∼22 nt regulatory RNA molecules that are involved in post-transcriptional regulation of gene expression through inhibition of translation initiation or targeting messenger RNAs for degradation [1], [2], [3]. miRNAs play important roles in regulation of numerous biological processes, such as development, cell proliferation and differentiation, metabolism, apoptosis, and the cell cycle [1], [4], [5], [6], [7]; hence, miRNAs are of medical importance. Moreover, alterations in miRNA regulation have been reported to be associated with several diseases such as cancer [8], [9], [10], [11].

Generally, the function of a miRNA is defined by its target genes and its effect on gene expression; thus, identification of potential miRNA targets is of highest importance. Since many potential miRNA regulated genes exist in a genome, accurate bioinformatic prediction is an essential tool for identifying targets. In animals, it is well known that miRNAs control gene expression through recognition of target sequences in the 3′-UTRs of mRNAs [4], [12]. Hence, efforts to predict target sites have often been limited to 3′-UTR regions, although occasionally other gene regions, e.g. 5′-UTR, CDS, were also considered. However, the currently available target information is not enough to fully explain the complete mechanism and function of miRNAs, especially the ones with unknown targets.

Recent experimental studies have suggested an alternative miRNA mechanism for modulating gene expression by targeting outside gene bodies through promoter recognition in human cells. Place et al. [13] provided evidence of promoter-targeting miRNA by showing that introduction of miR-373 induced expression of *CDH1* and *CSDC2* containing complementary promoter sequences. Kim et al. [14] reported the *cis*-regulatory role of miR-320 in targeting its own genomic location, which resulted in transcriptional silencing of an adjacent downstream gene, *POLR3D* through an AGO1-dependent mechanism. The recent report by Younger and Corey [15] demonstrated that miRNAs can silence gene expression by targeting gene promoters in *trans*. They showed that miRNAs with incomplete complementarity to their targets require AGO2 instead of AGO1, suggesting the possibility of different silencing mechanisms for different miRNA/target interactions. These studies indicated that the control of gene transcription by promoter targeting may be a general mechanism of miRNA gene regulation. Through promoter targeting, miRNAs may positively regulate gene expression [13] in a manner similar to RNA activation (RNAa) observed in synthetic dsRNAs targeting promoters [16], [17] or direct nuclear transcriptional gene silencing (TGS) [14], [15]. The underlying mechanism of regulation is still poorly understood and has not much been explored. Understanding this miRNA mechanism could lead to the development of a novel therapeutic approach for specifically controlling the expression of disease-associated target genes including the modulation of other phenotypes with associated expression profiles to promoter-targeting miRNAs.

So far, only a few miRNA-promoter interactions have been verified, possibly because there are few good candidate targets. A public resource providing genome-wide computationally identified potential miRNA binding sites on gene promoters could assist identification of biologically interesting candidates for validation [18], which is important given the paucity of experimental data for miRNA promoter targets. Despite a number of existing miRNA target databases such as miRDB [19], miRNAMap [20], miRGator [21], miRecords [22], the data of promoter targets are not readily available. To our knowledge, only recently developed miRWalk database [23] offers such data. However, the putative targets were only identified for sense strand and the detailed information of the predicted targets, which enables the screening of interesting candidates for experimental verification, is not provided.

We developed the microPIR (microRNA-Promoter Interaction Resource) database to facililate comprehensive exploration of putative miRNA binding sites within gene promoters of the human genome. In contrast to most currently available miRNA target databases, the predicted target data are managed in a way that enables the interactive and flexible exploration of putative binding sites that are most suitable with users' hypotheses, especially in the global view along with other associated genomic features and functions. The experimentally verified binding sites of AGO proteins, which have been reported to be involved in the mechanism of promoter-targeting miRNAs [14], [15], are incorporated as supporting information to enhance the effective screening of predicted target sites. The associated EST expression data including the sequence conservation data across vertebrate species of each predicted target are also incorporated into the database for the same purpose. Moreover, microPIR integrates various annotated genomic sequences, such as transposable elements (TEs) and repeats, transcription factor binding sites (TFBSs), CpG islands, and SNPs, allowing researchers to study relationships among target sites with these genomic features. All information can be visualized in a genome browser view format [24]. A local primer designing tool is incorporated into microPIR to assist experimental design of miRNA target validation. Target gene functional information is also available as link-outs from the microPIR search outputs.

## Results

**Figure 1:**
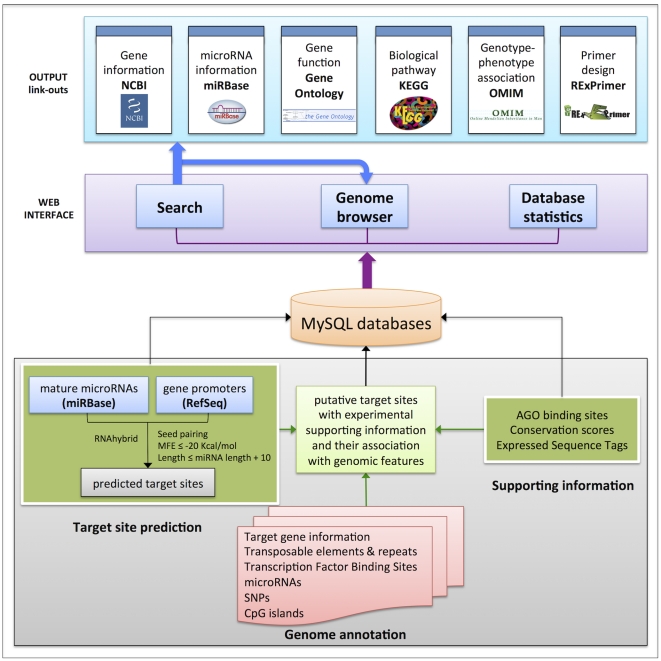
System overview of microPIR database. This is a three-tier system overview of the microPIR database displaying the data sources and web interface features.

microPIR presents miRNA target sites on promoter regions through a web interface with supporting features not only searching and retrieving data but also the visualizations of corresponding target sites. The web interface comprises three modules: 1) query, 2) genome browser, and 3) database statistics (Figure 1).

### Query module

**Figure 2:**
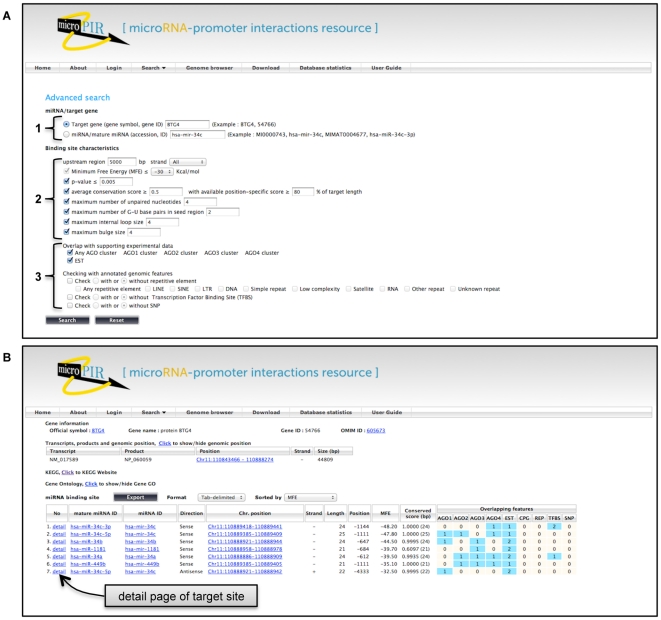
Search input and report output from the advanced search module. (A) The advanced search page is separated into three main parts. 1) A target gene or associated miRNA is put as a query. 2) The choices of binding site parameter settings can be adjusted to fit the user's needs. 3) The overlap of target site with specified annotated sequence is allowed as additional search criteria. (B) The list of resulting target sites obtained from search page is displayed. The information of associated miRNA is presented with the direction, chromosomal location, strand, length, upstream location, MFE, and conservation score of each target site including the number of bases with available score data. The number of different annotated sequences overlapping with each predicted target site is also shown. The hypertext link-outs to original sources of gene/miRNA associated information are provided. More target detail which includes a link to primer design is provided on the detail page of each target.

Two search options, basic and advanced, are provided for users to access target site information associated with the specified target gene or miRNA. The basic search supports inputs with the following types of identifiers: HUGO gene name, NCBI gene ID, miRNA accession and miRNA ID. Users can locate target sites within a certain gene by supplying a gene ID/symbol to the search form. Alternatively, a miRNA accession number/ID can be entered in order to view the list of target genes of this miRNA. This search option displays all target sites that pass the default parameter settings (see on the basic search page of web interface). The target site parameters can be customized to be more stringent with the advanced search option, which is designed to support diverse query types according to user's specific needs (Figure 2A). The adjustable parameters include strand orientation of target sites, the length of upstream region (default to 5000 bp), average conservation level of binding sites, miRNA-target binding pattern and parameters (Minimum Free Energy (MFE), *p*-value, number of unpaired nucleotides, G-U base pairs in seed region, internal loop size, and bulge size). The target output (s) can also be filtered based on the existence of supporting experimental information, AGO binding sites and/or EST, on predicted targets. The presence/absence of other annotated genomic sequences (TE and repetitive sequences, TFBSs, SNPs, CpG islands) in association with a target site can also be used as extra screening criteria as well.

The results obtained from the query page include the listing of target sites matched with the search criteria used (Figure 2B). This report shows the information of miRNA and target gene, genomic locations and length of the target sites, their positions relative to the transcription start sites of the target gene, strand (sense/antisense), MFE of miRNA-target binding and conservation level of target site. In addition, the presence/absence of different annotated sequences on each target is displayed. In the case of shared locations among different predicted targets of the same miRNA, the sites with the lowest MFE will be shown. The additional corresponding information is provided as hypertext link-outs, including gene information from NCBI gene database, gene function from Gene Ontology [25], gene biological pathways from KEGG [26], associated phenotypes from OMIM database (http://www.ncbi.nlm.nih.gov/omim), and miRNA information from miRBase [27]. More details on binding site including binding pattern and parameters are given with a link to the primer design service RExPrimer [28], which facilitates the design of corresponding primer pairs for the specific target site. The output can be sorted and exported as a text or BED file.

### Genome browser graphical representation

**Figure 3:**
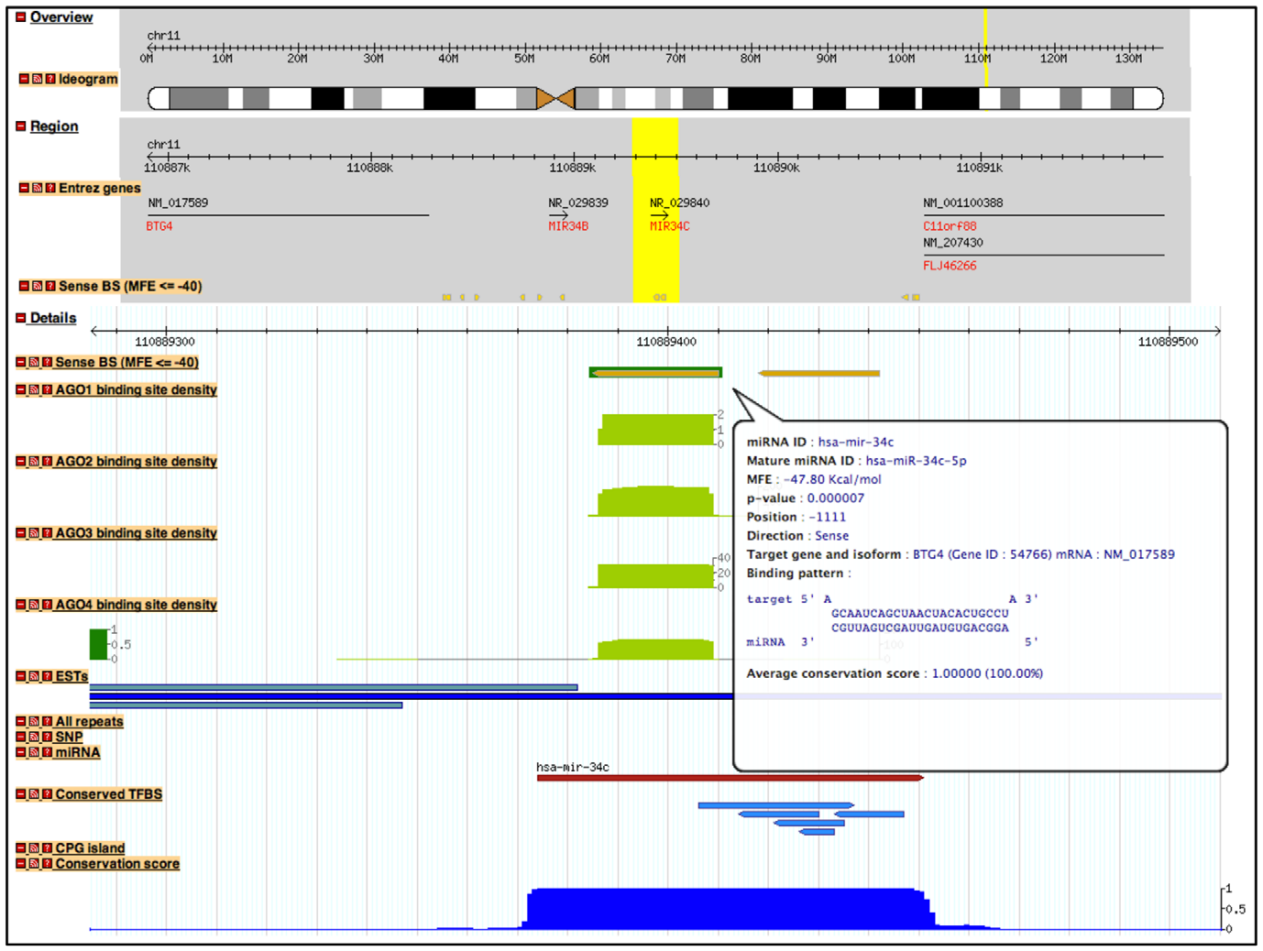
Genome browser displaying the resulting target sites with other genomic features. The position of target site is presented in an integrated view along with other supporting information and genomic annotations on a local genome broswer. The region-level view shows the distribution of putative target sites located within a specified genomic region. Users can highlight (yellow color) to zoom in the interested location for the detail-level view. The target site is displayed in gold box with green line. The conservation score of each nucleotide position (blue color on the bottom) is displayed in the range from 0 to 1. AGO binding-site cluster is represented as distribution of read numbers along the cluster (green color). In this particular case, the presence of miRNA (red color) on the same locus as its binding site represents the *cis*-regulatory role of miRNA.

Each target site can be visualized on a local genome browser (see Figure 3) implemented using a GBrowse generic browser service [24]. Through the customized viewing of the genome browser, users can visualize the position of a binding site within a gene promoter, as well as the spatial relationship between binding sites and other annotated sequences. Furthermore, the resolution of the genomic locus graphic can be adjusted from <100 bp to 1 Mbp. Certain supporting information can be displayed along with the location of target site (s). These features can be turned on/off at will.

### Database statistics

The summary information and initial analysis of data deposited in the microPIR database are displayed on the statistics page. The information includes build/release of miRNA/promoter sequences, number of unique targets, number of miRNA-target interactions, distribution of binding sites in terms of chromosomal locations, positions on the upstream regions, MFE, and *p*-value. The number of target sites found to be associated with each type of annotated sequence is also shown here. This information could guide researchers to make their decisions on searching parameters. This module will be automatically updated with the new release of microPIR.

## Discussion

We constructed a novel database which facilitates a comprehensive exploration of miRNA-promoter interactions. The genome-wide promoter target sites of miRNAs were predicted, organized, and integrated with a number of publicly available annotated sequence databases including the database of CLIP-generated AGO binding sites, gene and EST databases, database of vertebrate sequence conservation scores, genetic variation (SNP, repeat) databases, and gene regulation (TFBS, miRNA, CpG islands) databases. By incorporating these databases together with functional annotation, microPIR offers the most comprehensive and integrated view of these novel target sites.

Currently, the microPIR database contains a total of about 15 million unique target sites, with an approximately similar number of sense and antisense targets associated with 22,357 annotated genes. These numbers represent all possible sites on promoter sequences which pass the default parameter settings (perfect miRNA seed pairing, binding free energy ≤−20 Kcal/mol, and target length ≤ miRNA length+10) before considering any other parameter cutoff values. All experimentally verified cases of miRNA promoter targets [13], [14], [15] were detected and included in the database. The presence of putative miRNA targets on most of gene upstream sequences suggests that miRNA gene regulation through promoter targeting may be a more general event than expected. In stead of merely displaying all possible computational prediction results, microPIR supplies three major types of supporting genomic information, including genome map of experimentally verified AGO binding sites, ESTs and evolutionary conservation of miRNA target sites. It has been hypothesized that promoter-targeting miRNA regulated transcription occurs through recognition of noncoding RNA transcripts overlapping gene promoters [15]. In addition to protein-coding genes, a considerable number of noncoding transcripts originating from other genomic regions have been mapped in EST databases [29], and many of these noncoding transcripts overlap promoter regions. This observation supports the potentiality of predicted promoter targets presented in expressed sequences to be real target sites. AGO1/AGO2 proteins were demonstrated in two different studies to be recruited to the target promoter regions and were possibly involved in miRNA mechanism of action [14], [15]. Thus, AGO occupancy on predicted target sites can be used as an additional supported evidence for selection of target candidates. Also, evolutionarily conserved sequences would be more likely to be biologically functional miRNA target sites, since sequence conservation in promoter regions is generally lower than in gene coding regions [30], [31], [32]. This information is useful to prioritize target site candidates for further experimental study. For instance, with a specific set of target binding parameters (MFE≤−30 Kcal/mol, *p*-value≤0.005), a huge number of potential target sites can be narrowed down to 402 unique target candidates in 297 genes (211 genes are found in OMIM database), when considering only non-repeat derived, highly conserved sites (average conservation score ≥0.8) accompanied with experimental support, containing AGO1 or AGO2 binding sites, and are located in the expressed genomic region, as shown by EST data.

microPIR database was developed with the main purpose to provide a new data resource for miRNA researchers. Unlike other miRNA target databases, microPIR caters for a broad range of adjustable search options, giving a better control over various kinds of interactions interested by a user. For example, highly confident sites can be selected with the perfectly complementary binding sites by setting maximum number of unpaired nucleotide to be zero. Since it is generally accepted that endogenous miRNAs do not require complete complementarity to achieve target recognition, the stringency can also be adjusted to produce the corresponding targets. These flexible search options also allow the generation of hypotheses about possible miRNA/target relationships with other genomic elements, e.g. sequence polymorphisms, repetitive elements, TFBSs, CpG islands. Moreover, the genome browser view supports users' specific interest, e.g. researchers who are interested in the *cis*-regulatory role of promoter targeting miRNAs can look for target sites with their associated miRNA present in the same promoter region, as shown in Figure 3.

### Future development

Currently, microPIR provides information of miRNA-promoter target interactions for human sequences for which experimentally determined data of AGO binding sites and other genomic data are available. In the future, we plan to include data from more organisms in order to permit the comparative features across different species. Since the knowledge on miRNA-promoter interaction is in its early stage, the computational procedures for target site prediction will soon be updated. Additional computational prediction algorithms are planned to be used in the future. The scoring system of predicted target sites will be implemented and tested once enough cases of experimentally confirmed miRNA-promoter interactions are reported. To enhance the complete picture of the miRNA regulatory network, previously determined target sites within the body of genes will be included for further analysis.

### Conclusions

microPIR is the most comprehensive database specifically developed to provide an open access repository of information for miRNA target sites within human promoter sequences. Integration of considerable resources of genomic data and functional annotation allows researchers to investigate predicted miRNA-promoter interactions and generate new testable hypotheses and relavant biological insights of promoter-targeting miRNAs. The microPIR database is equipped with flexible search features including user-friendly GBrowse graphical interface and hypertext link-outs to various databases and a primer design service. We believe that microPIR will be a useful resource for the miRNA community to facilitate the discovery of new miRNA regulatory mechanism through promoter recognition.

## Materials and Methods

### microRNA target prediction

All known human miRNAs were downloaded from miRBase release 13.0 [27]. The 5000 bp upstream sequences of RefSeq genes were obtained from the UCSC Table Browser [33]. These sequences correspond with the hg18 assembly (human genome build 36). The promoter sequences were idenfied for the presence of putative miRNA target sites in both sense and antisense strands using the RNAhybrid program [34]. Complementarity to the miRNA seed sequence containing nucleotides 2–8 from the miRNA 5′end is well known as a major determinant for target recognition [1], [35], [36]. According to the most recent study [15], mutation of the seed sequence, but not other positions, abolished the silencing activity of promoter-targeting miRNA. As a minimum requirement, we set the desired target sequences to be completely complementary with the corresponding miRNA seed regions, the miRNA-target binding free energy ≤−20 Kcal/mol, and the target length ≤ miRNA length+10.

### Data resources and processing

Crosslinking and immunoprecipitation (CLIP) coupled with deep sequencing is a high-throughput experimental technique which has been applied to AGO proteins for identification of miRNA-target interactions [37]. We incorporated the data of 999350 CLIP-determined AGO binding sequences obtained from the CLIPZ database [38] as supporting information for predicted target sites. These AGO binding sites were mapped to human genome reference sequence hg18 (build 36.3). The sites that overlap by at least 1 bp were clustered, resulting in 1092430, 388859, 1966002 and 1317205 binding-site clusters for AGO1, AGO2, AGO3, and AGO4, respectively. The number of reads covering each nucleotide position in a cluster was counted. Each cluster is represented as the distribution of sequencing depth along the cluster.

Sequence conservation information of each predicted target site was also included into the database. Position-specific conservation scores, which were derived from multiple whole genome sequence alignments between the human and 16 other vertebrate genomes [39] were extracted from the UCSC Genome Browser [40]. These scoring values correspond to the posterior probability that a human genome site is conserved as computed by phastCons [41], and position-specific scores were averaged out across target sites to reflect the levels of evolutionary conservation among predicted target sites.

The GenBank human EST sequences were downloaded from the UCSC Table Browser [33]. In addition to transcript data, other genomic information was also included in microPIR. Transcription factor binding sites (6- to 12-nt consensus motif sequences) conserved in the human/mouse/rat alignment were retrieved from the UCSC Table Browser [33]. The genome locations and identities of transposable elements and repetitive elements were taken from annotations generated by the RepeatMasker program [42]. Finally, SNP data comprising more than nineteen million common and population-specific SNPs were obtained from various databases, namely NCBI dbSNP [43] build 129, HapMap [44] public release 27, JSNP [45] release 35, and ThaiSNP (http://www.biotec.or.th/thaisnp) release 2. The locations of CpG islands were taken from the UCSC Table Browser [33].

### System design and implementation

The overall system design of microPIR is illustrated in Figure 1. From the bottom of this figure, three data sources 1) target site prediction, 2) supporting genomic information for predicted targets, and 3) other genome annotations were preprocessed/collected and incorporated into the local MySQL databases. MySQL version 5.5.1 was employed to manage all the predicted target entries as well as their supporting information. To make the database accessible for public use, the web interface, including submission forms and graphical outputs were constructed using Python scripts and toolkits from Python Webware (http://www.webwareforpython.org). The web interface offers three main functionalities. First, the search feature allows users to locate miRNA target sites of interest. Data can be queried through the webform, which formulates the corresponding SQL queries to MySQL using python scripting language. We used Python MySQLdb module to connect (sending and receiving SQL queries/outputs) to the MySQL database backend. Second, the viewing feature enables users to graphically view the location of miRNA target sites within a specific locus via a genome browser interface. Third, the statistics module summarizes the information contained within this database for the genomic region of interest. The link-out module is provided for users to cross-check with other related databases, e.g. gene ontology and original data sources. Furthermore, for the sake of convenience, a primer design section is included to assist in validating potential target genes. The whole microPIR framework is running on our 12-core database server (2 AMD 6-core (2.8 GHz) processors with 64 Gigabytes of RAM and 2 Terabytes of hard disk space).
